# Status epilepticus resulted in rhabdomyolysis-induced AKI associated with hepatotoxicity induced by synergistic carbamazepine and diazepam: A case report

**DOI:** 10.1097/MD.0000000000036834

**Published:** 2024-02-23

**Authors:** Nawwar Soliman, Mohammad Alsultan, Ayham Alhusseini, Omar Alsamarrai, Kassem Basha

**Affiliations:** a Department of Internal Medicine, Al Assad and Al Mouwasat University Hospitals, Damascus University, Faculty of Medicine, Damascus, Syria; b Department of Nephrology, Al Assad and Al Mouwasat University Hospitals, Damascus University, Faculty of Medicine, Damascus, Syria; c Department of Neurology, Al Assad and Al Mouwasat University Hospitals, Damascus University, Faculty of Medicine, Damascus, Syria; d Nephrology Department, Al Mouwasat University Hospital, Damascus University, Faculty of Medicine, Damascus, Syria.

**Keywords:** acute kidney injury (AKI), acute liver injury (ALI), carbamazepine toxicity, rhabdomyolysis, status epilepticus (SE)

## Abstract

**Rationale:**

Rhabdomyolysis is a serious complication of status epilepticus (SE) caused by muscle cell damage and can lead to a life-threatening acute kidney injury (AKI).

**Patient concerns:**

A 35-year-old man with a history of seizures treated with 3 different antiepileptic drugs (carbamazepine, lamotrigine, and levetiracetam) presented with SE. The patient received 5 doses of diazepam to control the SE in another hospital and was transferred to our emergency due to AKI.

**Diagnoses:**

Laboratory tests corresponded with rhabdomyolysis-induced AKI and disseminated intravascular coagulation. Thereafter, the decrease in renal excretion of both drugs (diazepam and carbamazepine) caused acute liver injury and neurotoxicity. The carbamazepine concentration was 16.39 mcg/mL, which considered in toxic level, despite using the usual dose.

**Interventions:**

The patient was treated with hydration and sodium bicarbonate, however; severe AKI mandated a hemodialysis session.

**Outcomes:**

The diuresis started to increase, kidney and liver functions improved, and altered mental status reversed.

**Lessons:**

This case alerts physicians to consider the synergistic drug side effects and interactions, especially when patients present with impaired liver or kidney functions. The reduction in metabolism or excretion of drugs can cause an increase in serum concentrations and induce toxicity, even when the drug intake at the usual dose.

## 1. Introduction

Rhabdomyolysis is an emergency syndrome caused by skeletal muscle cell damage and the release of intracellular components such as potassium, creatine kinase (CK), myoglobin, and lactate dehydrogenase into the bloodstream.^[1]^ The causes of rhabdomyolysis are classified into traumatic and nontraumatic causes. Status epilepticus (SE) is considered one of the most important nontraumatic normal muscle causes of rhabdomyolysis.^[2,3]^

SE is a life-threatening neurological emergency defined as recurrent and prolonged seizures lasting more than 5 minutes without complete recovery of consciousness.^[4]^ SE needs to be diagnosed and treated rapidly, using antiepileptic drugs to control seizures.^[4]^

Carbamazepine is one of the antiepileptic drugs used to control seizures, treat neuropathic pain syndrome, and treat some psychiatric disorders. It is a cytochrome inducer and is metabolized by cytochrome 3A4 in the liver.^[5]^

Our patient was diagnosed with a rarely seen case of rhabdomyolysis-induced acute kidney injury (AKI) following SE, accompanied by carbamazepine toxicity.

## 2. Case report

A 35-year-old man was admitted to the emergency department in another hospital for 4 days because of recurrent generalized tonic-clonic seizures that lasted more than 30 minutes without recovery of consciousness between the episodes. During his admission, the patient received 5 ampules of intravenous diazepam to control his seizures. The patient was transferred to our hospital because of a continued loss of consciousness and a progressive decline in urine output and kidney function. The patient has a history of seizures after a car accident 20 years before and used carbamazepine, lamotrigine, and levetiracetam since then. In the family history, the mother has hypertension and diabetes mellitus type 2.

On admission, the patient was afebrile with no active seizures, and his blood pressure was 107/73 mm Hg, heart rate was 83 beats/minute and regular, respiratory rate was 24/minute, and saturation was 96% in room air.

The physical examination was unremarkable, and the neurologic examination showed: that the Glasgow Coma Scale was 12/15, the pupils were equal in size and reactive, refluxes were normal in the 4 limbs, and there were no signs of meningeal irritation or focal neurological deficit.

The initial laboratory tests and urine parameters are shown in Table 1. An electrocardiogram was performed and showed no abnormalities. A brain computed tomography scan showed no focal lesions, masses, or cerebral edema. The electroencephalogram did not reveal any epileptiform activity. An abdominal ultrasound showed normal size and shape with a slight cortical hyperdensity of both kidneys and normal liver appearance.

**Table 1:** The initial laboratory tests (December 12, 2021).

WBC		4.7		LDH	340 (H)		HBS AG		Neg	
Hb		13.2 (L)		CK	8266 (H)		Anti HCV		Neg	
PLT		127 (L)		TP	5.2 (L)		Anti HAV IgM		Neg	
Glu		98		Alb	3.4 (L)		Anri HAV IgG		Neg	
Ur		156 (H)		Ca	7.1 (L)		D.dimer		2.89 (H)	
Cr		5.24 (H)		P	6.7 (H)		Fib		125 (L)	
Na		120 (L)		PT	Prolonged		Carbamazepine concentration		16.39 (H)	
K		5.2 (H)		INR	Prolonged		PH		7.44	
Cl		80 (L)		TB	2.06 (H)		PCO2		26.9 (L)	
AST		1422 (H)		DB	0.56 (H)		HCO3		18.7 (L)	
ALT		2535 (H)		UA	12 (H)		AG		21.3 (H)	
Urine parameters										
Color	Clarity	PH	Specific gravity	RBCs	WBCs	Hb	Urate crystals	Protein		Bactria
Dark yellow	Turbide	5	1.020	90–100	9–10	+++	+++	+		++

AG = anion gab, Alb = albumin, ALP = alkaline phosphatase, anti HAV IgG = hepatitis A virus-specific IgG antibody, anti HAV IgM = hepatitis A virus-specific IgM antibody, anti HCV = anti hepatitis C virus antibodies, Ca = calcium, Cl = chloride, Cr = creatinine, DB = direct bilirubin, Glu = glucose, Hb = hemoglobin, HBS AG = hepatitis B surface antigens, HCO3 = serum bicarbonate, INR = international normalized ratio, K = potassium, Na = sodium, P = phosphor, PCO2 = partial pressure of CO2, Plt = platelets, PT = prothrombin time, TB = total bilirubin, TP = total proteins, UA = uric acid, Ur = urea, WBC = white blood cells.

AST: aspartate transaminase, normal range (8–33 U/L).

ALT: alanine transaminase, normal range (4–36 U/L).

LDH: lactate dehydrogenase, normal range (105–250 U/L).

CK: creatine kinase, normal range (55–170 U/L).

D.dimer: normal range (<0.5 mg/L).

Fib: fibrinogen, normal range (200–400 mg/dL).

At presentation, the patient had oliguria with impaired kidney and liver functions. The patient tests corresponded with AKI induced by rhabdomyolysis and acute live injury (ALI) with negative serologic tests for hepatitis. The ALI is suspected to be a result of hepatotoxicity caused by carbamazepine and/or diazepam. Furthermore, prolonged PT, thrombocytopenia, an elevated D-dimer level, and a low fibrinogen level suggested disseminated intravascular coagulation (DIC). The International Society on Thrombosis and Hemostasis (ISTH) score for DIC was 5,^[6]^ with no clinical signs of ischemic or bleeding events.

A peripheral blood smear showed a mild difference in size and shape of red blood cells (teardrop and target shapes), a normal white blood cell count, and a platelet count of 100 × 10*^3^/μL.

Symptomatic management was performed as follows: hydration with normal saline and 100 mEq of sodium bicarbonate intravenously, furosemide 60 mg/ daily to provoke diuresis, and a bolus of hypertonic saline to correct the hyponatremia, however, loss of consciousness continued unimproved.

Carbamazepine concentration was 16.39 mcg/mL (normal range is 4–12 mcg/mL), and a neurologist’s consultation suggested stopping carbamazepine and modifying doses of lamotrigine and levetiracetam.

After 4 days of admission, the patient developed anuria with progressive deterioration of kidney functions, as shown in Table 2. One hemodialysis (HD) session was performed with close monitoring of kidney functions and urine output. Urinary output started to increase, kidney function improved, and consciousness improved after 2 days of HD session (Table 2).

**Table 2:** Monitoring tests during admission.

Test	December 14, 2021 (day 2)	December 16, 2021 (day 4)	December 19, 2021 (day 7)
Ur	230	335*	126
Cr	6.7	8.19*	2.25
Na	126	129	133
AST	284	169	140
ALT	1305	301	298
CK	4300	–	325
LDH	1012	1211	201
PT	65%	95%	–
PLT	123	104	218
UA	–	7.7	3.3

* The HD session was performed.

The patient’s symptoms and laboratory tests gradually improved during the next 7 days of treatment, as shown in Table 2, and the patient was discharged after 2 weeks of admission.

## 3. Discussion

Status epilepticus (SE) is a neurological emergency defined as recurrent and prolonged tonic, clonic, or tonic-clonic movements of the extremities with no return to baseline.^[4]^ SE is categorized into convulsive and nonconvulsive status and the convulsive SE is classified into focal and generalized episodes.^[4,7]^ The most common causes of SE are structural lesions, infection, inflammation, and toxins.^[4,7]^ Genetic causes should be considered, especially at young ages.^[7]^

Rhabdomyolysis is a rare emergency complication of SE, defined as direct muscle cell damage and the release of intracellular components into the bloodstream.^[1]^ Rhabdomyolysis symptoms can range from mild symptoms like muscle pain and weakness with dark urine to a life-threatening condition with severe AKI.^[8,9]^ The most sensitive test to diagnose rhabdomyolysis is CK levels.^[3,8,9]^ CK rises during 2–12 hours after muscle cell damage and reaches its peak level within 24 to 72 hours.^[2]^ CK must increase at least 5 times over the upper limit of normal to diagnose rhabdomyolysis.^[3,8,10]^

Our patient presented with typical features of rhabdomyolysis when CK elevated nearly 50 folds from the upper limit of normal. Several metabolic abnormalities were observed, which included the following features:

- Elevated LDH, AST, and ALT; represent markers of muscle damage.
- Release of organic acids and intracellular components induced hyperuricemia, hyperkalemia, hyperphosphatemia, and anion gap metabolic acidosis.
- Hypocalcemia due to calcium sequestration in damaged muscle.
- Elevation of serum Cr levels.

AKI is a common complication of rhabdomyolysis with a frequency range of 15 to 50%.^[11]^ It has a very good prognosis, with a survival ratio of about 80%, and most patients recover to normal kidney function after treatment.^[11]^ About 27 to 37% of AKI complicated by rhabdomyolysis require HD.^[11]^ Multiple mechanisms would induce AKI in the context of rhabdomyolysis.^[3,8]^ The heme proteins released in the bloodstream from damaged muscles induce free radicals, which cause direct injury to the renal tubules and indirect injury by inducing vasoconstriction in renal vessels. Intravascular volume is depleted due to fluid sequestration in injured muscles. Also, obstructive intratubular cast formation is caused by the interaction between Tamm-Horsfall proteins and myoglobin, which is enhanced in acidic urine.^[2,8]^ Additionally, high uric acid generation provokes a high urinary excretion, in which results a tubular obstruction by uric acid casts.^[12]^

The treatment of AKI induced by rhabdomyolysis depends on early fluid resuscitation and inducing diuresis within the first 6 hours to minimize the risk of AKI.^[8,9]^ The goal is to increase renal perfusion and urinary flow rate to minimize tubular casts formation of uric acid and myoglobin, in addition to increasing potassium excretion.^[12]^ Also, bicarbonate and alkalinization of the urine are important to reduce cast formation by reducing the acidity of the urine.^[8,9]^ However, treatment with a large bicarbonate dose can enhance hypocalcemia, especially after fluid replacements.^[9]^

Our patient had no history of renal disease and presented with AKI after SE. The treatment included hydration with normal saline and 100 mEq/ daily of sodium bicarbonate for 4 days with close monitoring of electrolytes and urine output. Loop diuretics increase urinary flow and induce diuresis; however, their use in AKI prevention has also been debated.^[12]^ We try 60 mg of furosemide to induce diuresis in an attempt to convert oliguric to non-oliguric AKI and to lower the risk of needing HD.^[12]^ Unfortunately, anuria progressed after 4 days of admission with more deterioration of kidney function mandated HD. Likely, urinary output started to increase, kidney function improved, and no need for further HD sessions.

DIC is a rare and late complication of rhabdomyolysis, developing 12 to 72 hours after the injury.^[2,13]^ DIC during rhabdomyolysis is usually asymptomatic and caused by the activation of the coagulation cascade by the components of damaged cells.^[9]^ DIC is diagnosed depending on ISTH criteria^[6]^ and presents with thrombocytopenia, prolonged PT, and elevated D.dimer.^[9]^

Our patient’s tests were compatible with overt DIC (ISTH criteria was 5), and there were no ischemic or hemorrhagic features. The patient was treated for the underlying condition (rhabdomyolysis) and did not need any further transfusions based on treatment criteria for DIC.^[14]^

Carbamazepine has a wide use in internal medicine such as seizures and neuropathic pain syndromes.^[15]^ Carbamazepine is an inducer of cytochrome P450 and is metabolized via the liver cytochrome system to produce an active epoxide metabolite and it 72% of excretion via urine.^[5]^ Carbamazepine side effects include hepatotoxicity (about 30%) including hepatocellular hepatitis, cholestatic hepatitis, elevated liver functions, and hypersensitivity reactions. Also, the incidence of hyponatremia induced by carbamazepine was reported in about 1.8% to 40%.^[16]^ Carbamazepine toxicity occurs when the serum concentration is higher than 12 mcg/mL, especially with long-term use.^[17,18]^

Diazepam is a long-acting benzodiazepine used to control active seizures. Diazepam is metabolized by the cytochrome 3A4 system and is highly bound to plasma proteins (>95%).^[19]^ Diazepam metabolism is enhanced in patients taking long-term carbamazepine therapy. In some conditions, like hepatic impairment, the diazepam metabolism decreases, and the binding to plasma proteins of both diazepam and carbamazepine decreases, which increases the free drug concentrations in plasma and causes more side effects, especially hepatotoxicity and neurotoxicity.^[20]^ Additionally, diazepam overdose is frequently observed with the co-ingestion of other central nervous system depressants, which work synergistically and increase toxicity.^[21]^ Also, diazepam and its metabolites are excreted predominantly in the urine and accumulate upon multiple doses.^[21]^

**Figure 1:**
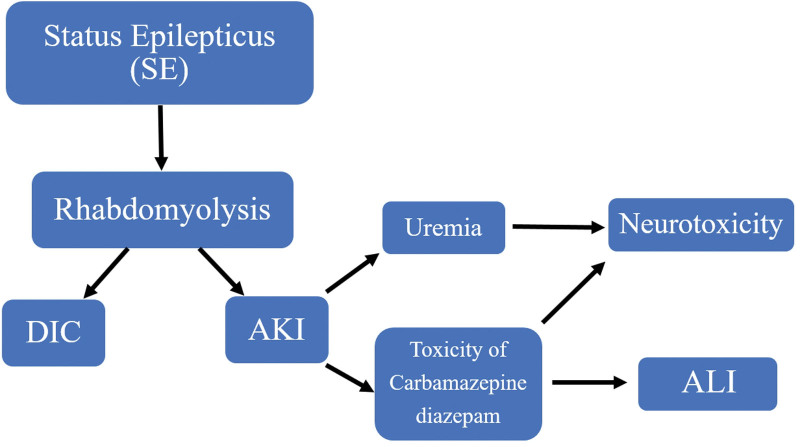
Timeline of the patients’ status: the priming event was SE. AKI further reduced the excretion of carbamazepine and diazepam, in which increased their levels. Toxic levels and side effects of carbamazepine and diazepam resulted in ALI. Eventually, neurotoxicity seems to be a result of uremia accompanied with synergistic effects of carbamazepine and diazepam. SE = status epilepticus; AKI = acute kidney injury, ALI = acute liver injury, DIC = diffuse intravascular coagulation.

In our patient, AKI induced by SE might cause a decrease in renal excretion of both drugs (diazepam and carbamazepine). This led to an increase in free drug concentrations and its metabolites, which enhanced hepatotoxicity and neurotoxicity. The carbamazepine concentration was 16.39 mcg/mL, which considered in toxic level, despite using the usual dose. This is clinically represented by ALI (elevated bilirubin and decreased albumin) and neurotoxicity (Glasgow Coma Scale = 12/15) (Fig. 1). Additionally, this theory was confirmed by the improvement of consciousness and liver markers after drug discontinuation and further drugs excretions in urine within days after improved kidney function. On the other hand, uremia could further worsen the mental status concomitant with previous drug effects (Fig. 1). As previously mentioned, rhabdomyolysis induced an elevation of ALT and AST levels, however, these high levels might result from synergistic and concomitant hepatocellular injury induced by diazepam and carbamazepine (Fig. 1).

## 4. Conclusions

This unusual and complicated case presented a patient with SE that was followed by rhabdomyolysis-induced AKI and DIC. Subsequently, AKI resulted in a decrease in renal excretion of both drugs (diazepam and carbamazepine), which caused ALI and neurotoxicity. Our report highlights the importance of an early diagnosis of rhabdomyolysis to prevent a life-threatening AKI. Physicians should consider the synergistic drug side effects and interactions, especially when patients present with impaired liver or kidney functions. The reduction in metabolism or excretion of drugs can cause an increase in serum concentrations and induce toxicity, even when the drug intake at the usual dose.

## Author contributions

**Conceptualization:** Nawwar Soliman, Ayham Alhusseini.

**Data curation:** Mohammad Alsultan.

**Investigation:** Mohammad Alsultan.

**Methodology:** Nawwar Soliman.

**Project administration:** Mohammad Alsultan.

**Software:** Omar Alsamarrai.

**Supervision:** Kassem Basha.

**Validation:** Nawwar Soliman, Omar Alsamarrai, Kassem Basha.

**Visualization:** Ayham Alhusseini, Omar Alsamarrai.

**Writing – original draft:** Nawwar Soliman, Mohammad Alsultan.

**Writing – review & editing:** Nawwar Soliman, Ayham Alhusseini, Omar Alsamarrai, Kassem Basha.
